# The impact of dose and discontinuation timing of preoperative ACE inhibitors on survival outcomes in cardiac surgery: A MIMIC-IV database analysis

**DOI:** 10.1371/journal.pone.0334889

**Published:** 2025-11-10

**Authors:** Xiu Zou, Shengyang Chen, Lingyan Hu, Lan Sha, Jianxing Zhou, Hongqiang Qiu, Xuemei Wu

**Affiliations:** 1 Department of Pharmacy, Fujian Medical University Union Hospital, Fuzhou, Fujian, China; 2 Department of Pharmacy, Fuwai Hospital, Chinese Academy of Medical Sciences, Beijing, China,; 3 Department of Pharmacy, The First Affiliated Hospital, Fujian Medical University, Fuzhou, Fujian, China; 4 Department of Pharmacy, National Regional Medical Center, Binhai Campus of the First Affiliated Hospital, Fujian Medical University, Fuzhou, Fujian, China; Scuola Superiore Sant'Anna, ITALY

## Abstract

**Background:**

Postoperative mortality following cardiac surgery remains high, highlighting the need to optimize perioperative medication strategies. Angiotensin-converting enzyme inhibitors (ACEIs) exert cardioprotective effects; however, the impact of their preoperative use on postoperative outcomes remains uncertain. This study evaluated the association between preoperative ACEI use and postoperative outcomes in cardiac surgery patients using a large dataset.

**Methods:**

This retrospective cohort study included patients from the MIMIC-IV database, grouped by preoperative ACEI use and non-ACEI use. Primary outcomes included in-hospital mortality; secondary outcomes included 30-day, 90-day, and 1-year mortality. Cox proportional hazards models were used to estimate hazard ratios (HR) and 95% confidence intervals (CI) for the association between ACEI use and postoperative mortality. Subgroup analyses were used to assess discontinuation timing and lisinopril doses. Propensity score matching was used to control for confounders.

**Results:**

Of 17,175 patients, 1,516 used ACEIs preoperatively. Cox modeling showed that preoperative ACEI use was significantly associated with reduced in-hospital mortality (HR 0.615; 95% CI 0.506–0.747; p < 0.001) and 30- and 90-day mortality (p < 0.05). Subgroup suggested a significant association between ACEI use and lower mortality in patients without malignancy. Continuing ACEIs on the day of surgery was associated with reduced in-hospital mortality, whereas discontinuation was associated with an attenuation of this beneficial association. A medium lisinopril dose (10–20 mg) was associated with the most consistent reduction in postoperative mortality. Results persisted after propensity score matching (p < 0.001).

**Conclusions:**

Continuing preoperative ACEI use on the day of surgery was associated with significantly reduced postoperative mortality. Medium-dose lisinopril was associated with the most consistent reduction in postoperative mortality, although patients with malignancy may require individualized assessment. These findings suggest that evidence-based perioperative ACEI management may be beneficial and warrant further investigation.

## 1. Introduction

Cardiac surgery remains a crucial treatment for various cardiac conditions. Despite significant advances in surgical techniques and perioperative care, adverse outcomes, including high postoperative mortality, continue to impact patient prognosis and quality of life substantially [1–3]. Consequently, refining perioperative management, particularly preoperative pharmacotherapy, is a key focus of clinical research. Angiotensin-converting enzyme inhibitors (ACEIs), mainstays in treating cardiovascular diseases, act primarily by inhibiting angiotensin II synthesis, which reduces vascular tone and blood pressure, decreases cardiac strain, and improves heart function [4,5]. Beyond these hemodynamic effects, ACEIs exhibit pleiotropic properties, including anti-inflammatory, antioxidant, and anti-fibrotic effects [6,7]. They suppress the NF-κB pathway and activate the Nrf2/HO-1 pathway, reducing pro-inflammatory cytokine production and mitigating myocardial ischemia-reperfusion injury [8,9]. Additionally, by enhancing nitric oxide bioavailability and reducing reactive oxygen species, ACEIs protect cardiac cells from oxidative stress [10]. They also inhibit transforming growth factor-β signaling, thereby reducing collagen deposition and cardiac fibrosis, which improves cardiac remodeling [11,12].

However, there is ongoing controversy about the use of ACEIs in the perioperative period following cardiac surgery, with a particular emphasis on the effect of preoperative use on postoperative results. Some research indicates that ACEIs may increase the risk of postoperative hypotension and acute kidney damage (AKI). These conditions can have a negative impact on the prognosis. For example, a prospective cohort study found that continuing to use ACEIs before non-cardiac surgery could result in intraoperative hypotension. This syndrome is connected with surgical consequences, including cardiovascular events and acute kidney injury [13]. A retrospective cohort study found that sustained preoperative usage of ACEIs increased the risk of intraoperative hypotension and postoperative myocardial damage [14]. Another study found that sustained preoperative usage of ACEIs significantly increased the incidence of intraoperative hypotension. This increased risk resulted in a larger demand for vasoactive drugs and a longer length of stay [15]. Given these contradictory findings, additional high-quality research is needed to further elucidate the relationship between ACEIs and postoperative outcomes in cardiac surgery. Current research on ACEIs and cardiac surgery prognosis frequently relies on small-sample RCTs with insufficient long-term follow-up data for broad populations. This study used MIMIC-IV data to investigate the relationship between preoperative ACEI use and postoperative mortality. Through this real-world study, we aim to provide more comprehensive evidence for optimizing preoperative medication management strategies in cardiac surgery.

## 2. Materials and methods

### 2.1. Data source

The present study employed a retrospective cohort design using the Medical Information Mart for Intensive Care IV (MIMIC-IV) database (version 2.2), which contains critical care information about patients admitted to the intensive care units at Beth Israel Deaconess Medical Center from 2008 to 2019. To maintain ethical standards and protect patient privacy, all datasets utilized in this study were de-identified, and necessary precautions were followed to ensure patient confidentiality. Ethical approval was not necessary because the study used de-identified publicly available data. Author Xiu Zou completed the National Institutes of Health’s online training course titled “Protecting Human Research Participants” (Record ID: 64,278,868) and was granted authorized access to the MIMIC-IV database for data extraction. Due to the retrospective nature and de-identified status of the data, the requirement for informed consent was waived by the Ethics Committee at Beth Israel Deaconess Medical Center.

### 2.2. Study population

Individuals who underwent cardiac surgery during hospitalization and met the following inclusion criteria were included in the study, as defined by the 9th and 10th Revisions of the International Classification of Diseases (ICD-9/10): 1) Length of stay > 24 h; 2) age > 18 years; 3) for patients who underwent multiple cardiac surgeries, only the record from the first surgery was included in the analysis.

### 2.3. Data extraction

Patient data were extracted from the MIMIC-IV database using structured query language (SQL) with PostgreSQL (version 16.4). This dataset included essential patient information, including demographic details, vital signs, laboratory test results, comorbidities, medication use, and clinical outcomes. Demographic information comprised age, gender, and ethnicity. Vital signs included respiratory rate, body temperature, heart rate, and blood pressure. Laboratory test results included metrics such as anion gap, blood urea nitrogen, glucose, and serum creatinine levels. Documented comorbidities included hypertension, diabetes mellitus, chronic kidney disease (CKD), heart failure, cancer, hyperlipidemia, and myocardial infarction. Medication information included whether ACEIs were used before cardiac surgery, the name of the ACEIs, doses, administration time, and discontinuation time. ACEI discontinuation timing was determined using exact medication administration timestamps from the MIMIC-IV database’s “inputevents” table. Discontinuation was categorized as: (1) “discontinued the day before surgery” when the last dose was administered >24 hours before surgery start time, or (2) “discontinued on the day of surgery” when the last dose was given ≤24 hours before surgery (including all administrations occurring on the calendar day of surgery, irrespective of exact timing).

### 2.4. Outcomes

The primary outcome of our study was in-hospital mortality, and the secondary outcomes were 30-day, 90-day, and 360-day mortality after admission.

### 2.5. Statistical analysis

Continuous variables are reported as mean ± standard deviation (SD) or as median (interquartile range, IQR). The differences between groups were analyzed using either the t-test or the Mann-Whitney U test. Categorical variables are presented as frequencies (percentages) and were compared between groups using the chi-square test, corrected chi-square test, or Fisher’s exact test. Variables with <25% of missing data were included in the analysis. Missing data were handled using multiple imputation (MICE) with random forest in R, generating five imputed datasets. All analysis variables were included, and results were pooled using Rubin’s rules. Multicollinearity was assessed via variance inflation factors (all VIFs < 2), confirming negligible collinearity.

A Cox proportional hazards model was utilized to calculate the hazard ratio (HR) and the corresponding 95% confidence interval (CI) for preoperative use of ACEIs. Adjustments for multiple variables were performed. Model 1 was unadjusted; Model 2 was adjusted for gender, age, and race; Model 3 was built on Model 2 and further adjusted for respiratory rate, temperature, heart rate, blood pressure, anion gap, blood urea nitrogen, glucose, serum creatinine, CKD, myocardial infarction, hypertension, diabetes mellitus, heart failure, and hyperlipidemia. Additionally, we conducted subgroup analyses stratified by age, gender, race, and specific conditions such as CKD, myocardial infarction, hypertension, diabetes mellitus, heart failure, and hyperlipidemia to explore the association between the preoperative use of ACEIs and outcomes in cardiac surgery patients and to investigate potential interactions. We compared postoperative mortality differences between three distinct groups of cardiac surgery patients: those who discontinued ACEIs the day before surgery, those who discontinued ACEIs on the day of surgery, and those who did not discontinue ACEIs at all. We analyzed the dose effect of preoperative lisinopril use on reducing postoperative mortality in cardiac surgery patients. We conducted sensitivity analyses to verify the robustness of the study results. To balance baseline differences between the postoperative mortality and non-mortality groups, we performed propensity score matching (PSM) using a logistic regression model incorporating 17 clinically relevant covariates: age, gender, race, anion gap, blood urea nitrogen, glucose, serum creatinine, malignancy, respiratory rate, heart rate, temperature, CKD, diabetes mellitus, myocardial infarction, heart failure, hypertension, and hyperlipidemia. We employed 1:1 nearest neighbor matching, applying a caliper width of 0.1 standard deviations of the logit propensity score to ensure high-quality matches. The matching process achieved a satisfactory balance, with post-matching standardized mean differences (SMD) < 0.10 for all covariates. Subsequent analyses were conducted on the matched cohort to enhance comparability between groups.

All data analyses were conducted through R version 4.2.2. A two-sided *p*-value of < 0.05 was considered statistically significant.

## 3. Result

### 3.1. Patient selection and baseline characteristics

**Fig 1:**
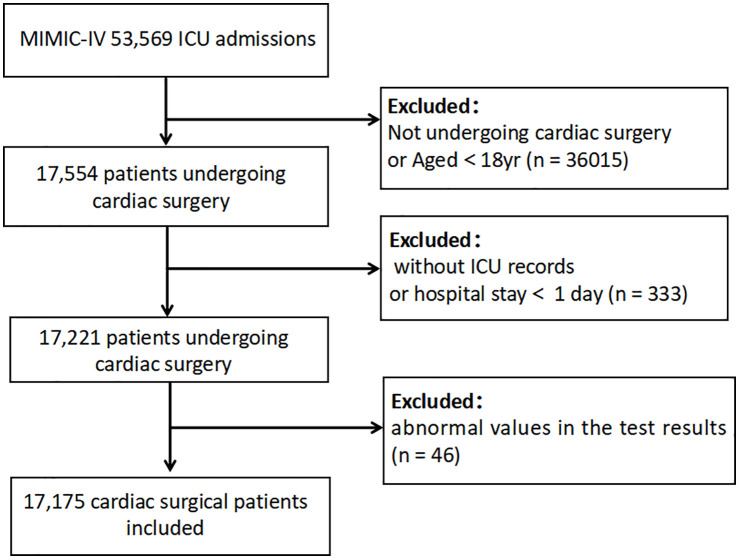
Flowchart of patient selection for the study. ICU: intensive care unit; MlMlC-lV: Medical Information Mart for Intensive care Database lV.

As shown in Fig 1, 17,175 patients were enrolled in this study. They were divided into two groups: the ACEI group, which included 1,516 patients who used ACEIs before cardiac surgery, and the non-ACEI group, which included 15,659 patients who did not use ACEIs. The in-hospital mortality was 1,820 patients (10.60%). The median age of patients undergoing cardiac surgery was 67.42 years. The median ages of patients who used ACEIs preoperatively and who did not use ACEIs were 69.97 and 67.15 years, respectively. The proportion of patients with cancer was lower in the ACEI group than in the non-ACEI group (11.1% vs. 14.2%). However, the ACEI group had higher proportions of patients with CKD (25.9% vs. 22.6%), diabetes mellitus (46.7% vs. 34.1%), myocardial infarction (31.2% vs. 21.8%), heart failure (46.2% vs. 29.7%), hypertension (68.5% vs. 51.4%), hyperlipidemia (53.9% vs. 42.6%), and male patients (61.5% vs. 57.2%) (Table 1).

**Table 1:** Characteristics of patients included in the study from the MIMIC-IV (2008 to 2019) database.

Characteristic	Overall	Non-ACEI users	ACEI users	*p*-value
	N = 17,175	N = 15,659	N = 1,516	
Gender (%)				0.001
Male	9,896 (57.6)	8,963 (57.2)	933 (61.5)	
Female	7,279 (42.4)	6,696 (42.8)	583 (38.5)	
Admission age (median [IQR])	67.42 [56.59, 78.20]	67.15 [56.28, 78.05]	69.97 [60.81, 79.27]	<0.001
Race (%)				0.041
Asian	467 (2.7)	438 (2.8)	29 (1.9)	
Black	1,445 (8.4)	1,302 (8.3)	143 (9.4)	
Other	3,310 (19.3)	2,997 (19.1)	313 (20.6)	
Whtie	11,953 (69.6)	10,922 (69.7)	1,031 (68.0)	
Total hospital length of stay (median [IQR])	8.00 [5.02, 13.76]	7.83 [4.91, 13.52]	10.20 [6.98, 15.28]	<0.001
In-hospital mortality indicator (%)	1,820 (10.6)	1,709 (10.9)	111 (7.3)	<0.001
Intensive Care Unit length of stay (median [IQR])	2.32 [1.30, 4.73]	2.33 [1.30, 4.78]	2.24 [1.28, 4.25]	0.033
Anoin gap (median [IQR])	14.00 [12.00, 16.00]	14.00 [12.00, 16.00]	14.00 [12.00, 16.00]	0.039
BUN (median [IQR])	19.00 [14.00, 29.00]	19.00 [14.00, 29.00]	21.00 [15.00, 30.00]	<0.001
Glucose (median [IQR])	113.00 [96.00, 143.00]	113.00 [95.00, 143.00]	120.00 [99.00, 151.00]	<0.001
Scr (median [IQR])	1.00 [0.80, 1.30]	1.00 [0.80, 1.30]	1.00 [0.80, 1.30]	<0.001
LVEF (median [IQR])	55.00 [45.00, 55.00]	55.00 [45.00, 55.00]	55.00 [40.00, 55.00]	<0.001
RR (median [IQR])	17.00 [14.00, 21.00]	17.00 [14.00, 21.00]	17.00 [14.00, 21.00]	0.201
Temperature (median [IQR])	98.00 [97.40, 98.70]	98.00 [97.40, 98.70]	98.10 [97.50, 98.70]	0.133
HR (median [IQR])	84.00 [74.00, 98.00]	84.00 [74.00, 98.00]	83.00 [74.00, 96.00]	0.006
Cancer (%)	2,397 (14.0)	2,228 (14.2)	169 (11.1)	0.001
Chronic kidney disease (%)	3,924 (22.8)	3,532 (22.6)	392 (25.9)	0.004
Diabetes mellitus (%)	6,040 (35.2)	5,332 (34.1)	708 (46.7)	<0.001
Myocardial infarction (%)	3,891 (22.7)	3,418 (21.8)	473 (31.2)	<0.001
Heart failure (%)	5,356 (31.2)	4,656 (29.7)	700 (46.2)	<0.001
Essential hypertension (%)	9,079 (52.9)	8,041 (51.4)	1,038 (68.5)	<0.001
Hyperlipidemia (%)	7,491 (43.6)	6,674 (42.6)	817 (53.9)	<0.001
Surgery Category, n(%)				<0.001
Vascular surgery	13570 (79.01)	12452 (79.52)	1118 (73.75)	
Valve and pericardial Surgery	3474 (20.23)	3080 (19.67)	394 (25.99)	
Other	131 (0.76)	127 (0.81)	4 (0.26)	

ACEI: Angiotensin-converting enzyme inhibitor; IQR: interquartile range; BUN: Blood Urea Nitrogen; Scr: Serum Creatinine; LVEF: Left Ventricular Ejection Fraction; RR: Respiratory Rate; HR: Heart Rate.

### 3.2. Association between ACEIs and all-cause mortality

This study evaluated the association between the preoperative use of ACEIs and postoperative mortality through a Cox proportional hazards model. The model was adjusted stepwise for various covariates. As shown in Supporting Information, S1 Table, the preoperative use of ACEIs was significantly associated with reduced in-hospital mortality in the unadjusted model (Model 1) (HR = 0.565, CI: 0.466–0.685, *p* < 0.001), the model adjusted for age, gender, and race (Model 2) (HR = 0.508, CI: 0.419–0.616, *p* < 0.001), and the model further adjusted for all clinical and laboratory variables (Model 3) (HR = 0.615, CI: 0.506–0.747, *p* < 0.001).

Additionally, the study analyzed the association between the preoperative use of ACEIs and 30-day, 90-day, and 360-day mortality. Results showed that in Model 1, the preoperative use of ACEIs was associated with reduced 30-day (HR = 0.669, *p* < 0.001), 90-day (HR = 0.791, *p* < 0.001), and 360-day mortality (HR = 0.875, *p* = 0.017). In Model 2, the association was further strengthened for 30-day (HR = 0.614, *p* < 0.001), 90-day (HR = 0.725, *p* < 0.001), and 360-day mortality (HR = 0.804, *p* < 0.001). In Model 3, 30-day (HR = 0.731, *p* < 0.001) and 90-day (HR = 0.845, *p* < 0.05) mortalities remained significantly reduced. The preoperative use of ACEIs was significantly associated with reduced postoperative 30-day, 90-day, and 360-day mortality, and this association remained significant even after adjustment for covariates.

### 3.3. Survival curve analysis

**Fig 2:**
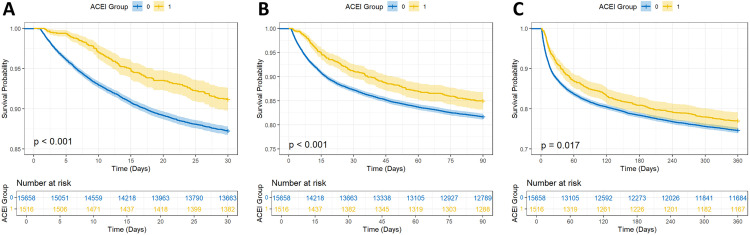
Kaplan-Meier survival curves were used to analyze the mortality rates of cardiac surgery patients with preoperative ACEIs use over three time points: 30-day (A), 90-day (B), and 360-day(C). 0: non-ACEI group; 1: ACEI group.

Survival analysis was conducted to compare 30-day, 90-day, and 360-day survival rates between the two groups before and after matching. Kaplan-Meier survival analysis showed that the survival rates at 30, 90, and 360 days were significantly higher in the ACEI group than those in the non-ACEI group (log-rank test: *p* < 0.05) (Fig 2).

### 3.4. Subgroup analysis

**Fig 3:**
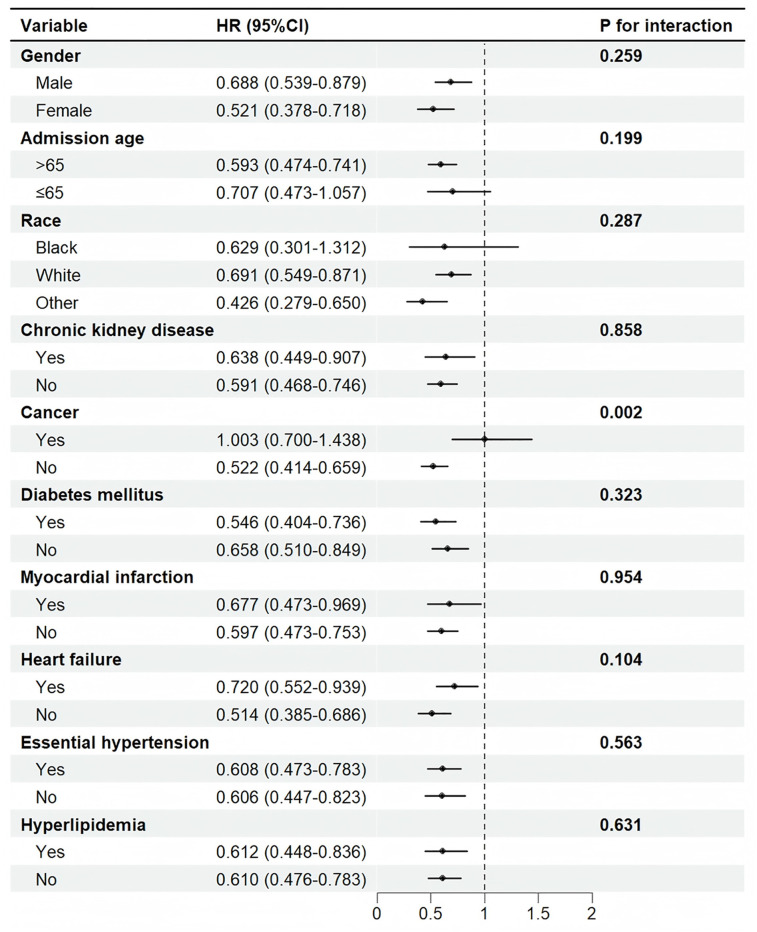
Subgroup analysis of the relationship between ACEIs use and in-hospital mortality. HR: Hazard Ratio, CI: confidence interval.

As shown in Fig 3, the preoperative use of ACEIs was associated with reduced postoperative mortality across various subgroups, including age, gender, race, malignancy, CKD, diabetes mellitus, myocardial infarction, heart failure, hypertension, and hyperlipidemia. This association was more pronounced in patients over 65 years and among Caucasians and other racial groups (*p* < 0.001). No significant interaction was observed between these variables and ACEIs (*p* for interaction > 0.05). In the malignancy subgroup, the use of ACEIs was significantly associated with reduced in-hospital mortality risk in patients without malignancy (HR = 0.522, *p* < 0.001). Conversely, this association was not significant in patients with malignancy (HR = 1.003, *p* = 0.986). A significant interaction between malignancy and the use of ACEIs on postoperative mortality was observed (*p* for interaction = 0.002).

### 3.5. Preoperative discontinuation analysis

ACEI users were categorized based on discontinuation time: discontinued 1 day before surgery (613 patients), discontinued on the day of surgery (494 patients), and not discontinued (409 patients). As shown in Supporting Information, S2 Table, there was a significant association between ACEI discontinuation time and patient mortality. In terms of in-hospital mortality, compared to the non-ACEI group, patients who did not discontinue ACEIs had the lowest risk (HR = 0.48, *p* < 0.001), followed by those who discontinued on the day of surgery (HR = 0.574, *p* = 0.005) and those who discontinued 1 day before surgery (HR = 0.711, *p* = 0.009). This suggests that continuing ACEIs preoperatively may benefit in reducing in-hospital mortality.

As presented in Supporting Information, S3 Table, for 30-day mortality, the risk was significantly lower in the groups that discontinued on the day of surgery (HR = 0.623, *p* = 0.006) and did not discontinue (HR = 0.635, *p* = 0.011) than that in the non-ACEI group, although there was no significant difference between the group that discontinued 1 day before surgery (*p* = 0.254). The results for 90-day mortality were similar; a significant risk reduction was observed in the group that discontinued on the day of surgery (HR = 0.666, *p* = 0.004)(Supporting Information, S4 Table). For 360-day mortality, only the group that discontinued on the day of surgery showed a significant risk reduction (HR = 0.755, *p* = 0.011); there were no significant differences observed in the other groups(Supporting Information, S5 Table).

### 3.6. Dose analysis

To further explore the association between doses of ACEIs and postoperative mortality in cardiac surgery patients, this study analyzed the medication data of the 1,516 patients who used ACEIs preoperatively. Among them, 7, 123, 72, 12, 1,279, and 22 used benazepril, captopril, enalapril maleate, enalaprilat, lisinopril, and ramipril, respectively. Considering the different standard doses for various ACEIs, this study focused on the effects of lisinopril, an ACEI commonly used in clinical practice, on in-hospital and long-term mortality in cardiac surgery patients. To further investigate the impact of drug dose on mortality, lisinopril was categorized into high-dose (> 20 mg), medium-dose (10–20 mg), and low-dose (<10 mg) groups based on clinical medication practices. There were 501, 300, and 478 patients in the high-, medium-, and low-dose groups, respectively. As shown in Supporting Information, S6 Table, lisinopril use was associated with a significant reduction in in-hospital mortality, and there was a clear dose-response relationship. For in-hospital mortality, all dose groups showed a significant risk reduction (*p* < 0.05). This indicates a significant association between lisinopril use and reduced in-hospital mortality. Regarding 30-day mortality, the medium-dose (10–20 mg) and high-dose (≥ 20 mg) groups showed a significant risk reduction (*p* < 0.05), whereas the low-dose group did not (*p* > 0.05)(Supporting Information, S7 Table). For 90-day and 360-day mortality, the medium-dose (10–20 mg) group continued to show a significant risk reduction (*p* < 0.05), while the high-dose group exhibited a significant effect at 90 days (*p* < 0.05) but not at 360 days (*p* > 0.05). Overall, the medium-dose (10–20 mg) group demonstrated the most stable and significant performance in reducing postoperative mortality (Supporting Information, S8, S9 Tables).

### 3.7. Sensitivity analysis

The study used PSM to match the original data in a 1:1 ratio. This process resulted in the formation of 3,610 pairs of matched patients. The association between the preoperative use of ACEIs and postoperative mortality was reevaluated. As shown in Supporting Information, S10 Table, after matching, patients who used ACEIs preoperatively had significantly lower in-hospital, 30-day, 90-day, and 360-day mortality than those in non-users (*p* < 0.001). Compared to the results before matching, the protective effect of ACEIs remained significant and robust after matching. This indicates that ACEIs are still significantly associated with reduced postoperative mortality, even when differences in baseline characteristics are controlled. PSM further validated the protective effect of ACEIs in cardiac surgery patients, suggesting that using ACEIs as a preoperative medication may help improve postoperative survival rates.

## 4. Discussion

This study conducted a retrospective cohort analysis to investigate the association between the preoperative ACEI use and postoperative mortality in cardiac surgery patients. The results showed that the preoperative use of ACEIs was associated with a significant reduction in in-hospital, 30-day, 90-day, and 360-day mortality. After adjustments were performed for various confounding factors, this association remained robust, suggesting the potential value of ACEIs in improving the prognosis of patients undergoing cardiac surgery.

Our findings align with previous research supporting the prognostic benefit of ACEIs, likely mediated through their multifaceted mechanisms [16–18]. For example, one meta-analysis demonstrated a 22% reduction in in-hospital/30-day mortality among ACEI users [16]. Zhang et al. [17] investigated the clinical outcomes of the perioperative use of ACEIs in heart failure (HF) patients undergoing Coronary Artery Bypass Grafting (CABG) or valve surgery and found that the preoperative use of ACEIs reduced all-cause mortality (HR = 0.70) and major adverse cardiovascular and cerebrovascular events (HR = 0.76). According to a randomized controlled research, stopping ACEIs before surgery may prevent some high-risk groups receiving CABG, such as patients with metabolic syndrome, from benefiting from the organ-protective effects of ACEIs [18]. These results are consistent with the complex mechanisms of ACEIs. These pathways include antioxidant, anti-inflammatory, anti-myocardial fibrosis, and actions that promote endothelial function. These diverse mechanisms imply that ACEIs may improve hemodynamic stability, lower systemic inflammatory response, and prevent myocardial remodeling to have cardioprotective benefits before surgery [19–21]. Consequently, ACEIs can reduce postoperative complications and enhance cardiac surgery outcomes.

Subgroup analysis revealed a significant interaction between ACEI treatment and malignancy status regarding postoperative mortality. While ACEI use was significantly associated with reduced in-hospital mortality risk in patients without malignancy (HR = 0.522, p < 0.001), no significant association was observed in patients with malignancy (HR = 1.003, p = 0.986). This neutral finding suggests that the potential benefits of ACEIs observed in the overall population may not extend to patients with active cancer. These results indicate that for cardiac surgery patients with malignancy, the risk-benefit ratio of perioperative ACEI use requires careful consideration, and individualized decision-making is warranted.

There are notable differences in current study findings and recommendations regarding the subject of whether renin-angiotensin system inhibitors should be stopped before major surgery [22–24]. The STOP-or-NOT trial, a large multicenter randomized trial, attempts to resolve this controversy; nonetheless, the results are not yet conclusive [25]. Continued perioperative use of ACEIs, either stopping on the day of surgery or not stopping at all, was linked to significantly lower in-hospital and long-term mortality, according to our study’s examination of preoperative cessation. According to a multicenter observational study, continuing ACEIs on the day of surgery did not raise the risk of postoperative AKI or the incidence of major cardiovascular events. This finding is in line with that study [26]. By preserving the homeostasis of the RAAS system and reducing inflammation and vasospasm, prolonged usage may have beneficial effects. Surgical stress causes both vasospasm and inflammatory reactions. Significantly, the benefit was lessened in the group that stopped using ACEIs one day before surgery, indicating that the medication’s protective benefits might be time-dependent and that stopping it too soon could lessen its organ-protective benefits [27,28]. As a result, maintaining ACEIs before surgery may assist in preserving their cardioprotective benefits, lower postoperative risks, and enhance patient outcomes. This offers provides crucial observational evidence to inform ACEI medication timing optimization in clinical practice.

The results of the dose study demonstrated a distinct dose-response connection between postoperative mortality and various lisinopril dosages. While the benefit of the high-dose group waned over time, the medium-dose group (10–20 mg) showed the most consistent and noteworthy decrease in postoperative mortality. This suggests that optimizing the ACEI dose is crucial for improving outcomes, as excessively high doses may not confer additional benefits and could increase the risk of adverse effects [29]. Therefore, clinicians should consider the dose-response relationship and individual patient factors when prescribing ACEIs.

This study has important clinical implications. First, it clarifies that continuing ACEIs until the day of cardiac surgery can significantly reduce short-term and long-term postoperative mortality. This provides observational evidence that supports further research into perioperative medication strategies. Second, the dose analysis results suggest that clinicians should adjust the dose of ACEIs based on the patient’s specific situation to achieve the best long-term protective effect. Notably, this study revealed a significant protective effect of ACEIs in patients without malignancy but limited effects in patients with malignancy. This finding suggests that clinicians need to individualize medication regimens based on patient comorbidities. These findings will directly optimize perioperative management of cardiac surgery patients and improve clinical outcomes. However, this study has several limitations that should be acknowledged. First, as a retrospective observational study using data from the single-center MIMIC-IV database, our findings may be susceptible to selection bias and unmeasured confounding despite our robust statistical adjustments. The generalizability of our results may be limited by this single-center source, and they require validation in multicenter and prospective cohorts before any clinical recommendations can be considered. Second, while the MIMIC-IV database provides comprehensive clinical information, the high rate of missing data for some variables may affect the completeness. Third, and importantly, the absence of detailed intraoperative data represents a significant limitation. We lacked information on surgical complexity, specific procedural details, hemodynamic events, and anesthetic management, all of which may strongly influence postoperative outcomes and could potentially confound the observed associations. Fourth, our dose-response findings are specific to lisinopril and may not generalize to other ACE inhibitors due to pharmacological differences. Finally, while propensity score matching and multivariable adjustment were employed, residual confounding from unmeasured factors cannot be entirely excluded.

In conclusion, this study, through large-sample data analysis, confirmed that the preoperative use of ACEIs is significantly associated with reduced postoperative mortality, especially in specific patient groups. Future research should include multicenter data and further optimize the use of ACEIs in different types of surgeries and patient populations to optimize perioperative management with the goal of improving patient outcomes.

## Supporting Information

S1 Table Association between preoperative use of ACEIs and all-cause mortality. (DOCX)

S2 Table Relationship between preoperative discontinuation of ACEIs and in-hospital mortality in cardiac surgery patients. (DOCX)

S3 Table Relationship between preoperative discontinuation of ACEIs and 30-day postoperative mortality in cardiac surgery patients. (DOCX)

S4 Table Relationship between preoperative discontinuation of ACEIs and 90-day postoperative mortality in cardiac surgery patients. (DOCX)

S5 Table Relationship between preoperative discontinuation of ACEIs and 360-day postoperative mortality in cardiac surgery patients. (DOCX)

S6 Table Dose-response relationship between preoperative lisinopril use and hospital mortality in cardiac surgery patients. (DOCX)

S7 Table Dose-response relationship between preoperative lisinopril use and 30-day mortality in Cardiac Surgery Patients. (DOCX)

S8 Table Dose-response relationship between preoperative lisinopril use and 90-day mortality in Cardiac Surgery Patients. (DOCX)

S9 Table Dose-response relationship between preoperative lisinopril use and 360-day mortality in Cardiac Surgery Patients. (DOCX)

S10 Table Association between preoperative use of ACEIs and all-cause mortality after propensity score matching. (DOCX)
