# Supplementary material for: The impact of dose and discontinuation timing of preoperative ACE inhibitors on survival outcomes in cardiac surgery: A MIMIC-IV database analysis
Source: PLoS One. 2025 Nov 10;20(11):e0334889. doi: 10.1371/journal.pone.0334889 (PMC12599911; doi:10.1371/journal.pone.0334889)
Supplement: S1 Table — (DOCX) [file pone.0334889.s001.docx]

| **Table S1** Association between preoperative use of ACEIs and all-cause mortality | | | | | | |
| --- | --- | --- | --- | --- | --- | --- |
| Variables | Model 1 | | Model 2 | | Model 3 | |
|  | HR (95% CI) | *p*-value | HR (95% CI) | *p*-value | HR (95% CI) | *p*-value |
| In-hospital mortality |  | | | | | |
| ACEIs vs. Non-ACEIs | 0.565 (0.466, 0.685) | <0.001 | 0.508 (0.419, 0.616) | <0.001 | 0.615 (0.506, 0.747) | <0.001 |
| 30-day mortality |  | | | | | |
| ACEIs vs. Non-ACEIs | 0.669 (0.562, 0.797) | <0.001 | 0.614 (0.515, 0.731) | <0.001 | 0.731 (0.613, 0.872) | <0.001 |
| 90-day mortality |  | | | | | |
| ACEIs vs. Non-ACEIs | 0.791 (0.691, 0.905) | <0.001 | 0.725(0.634, 0.830） | <0.001 | 0.845 (0.737, 0.969) | 0.016 |
| 360-day mortality |  | | | | | |
| ACEIs vs. Non-ACEIs | 0.875 (0.784, 0.976) | 0.017 | 0.804(0.721, 0.897) | <0.001 | 0.911 (0.816, 1.018) | 0.099 |
| Model 1: Unadjusted.  Model 2: Adjusted for gender, age, race.  Model 3: Adjusted for gender, admission age, race, anion gap, bun, glucose, scr, cancer, rr, chronic kidney disease, diabetes mellitus, temperature, myocardial infarction, hr, heart failure, essential hypertension, hyperlipidemia.  ACEI, Angiotensin-converting enzyme inhibitor; CI, confidence interval; HR, hazard ratio. | | | | | | |
