# Supplementary material for: The impact of dose and discontinuation timing of preoperative ACE inhibitors on survival outcomes in cardiac surgery: A MIMIC-IV database analysis
Source: PLoS One. 2025 Nov 10;20(11):e0334889. doi: 10.1371/journal.pone.0334889 (PMC12599911; doi:10.1371/journal.pone.0334889)
Supplement: S3 Table — (DOCX) [file pone.0334889.s003.docx]

| **Table S3** Relationship between preoperative discontinuation of ACEIs and 30-day postoperative mortality in cardiac surgery patients | | | |
| --- | --- | --- | --- |
| Group | HR | 95% CI | *p*-value |
| Non | 1 | - | - |
| Discontinued 1 day preoperatively | 0.868 | 0.680–1.107 | 0.254 |
| Discontinued on day of surgery | 0.623 | 0.443–0.875 | 0.006 |
| Continued through surgery | 0.635 | 0.448–0.902 | 0.011 |
| ACEIs, Angiotensin-converting enzyme inhibitors; CI, confidence interval; HR, hazard ratio. | | | |
