# Supplementary material for: The impact of dose and discontinuation timing of preoperative ACE inhibitors on survival outcomes in cardiac surgery: A MIMIC-IV database analysis
Source: PLoS One. 2025 Nov 10;20(11):e0334889. doi: 10.1371/journal.pone.0334889 (PMC12599911; doi:10.1371/journal.pone.0334889)
Supplement: S4 Table — (DOCX) [file pone.0334889.s004.docx]

| **Table S4** Relationship between preoperative discontinuation of ACEIs and 90-day postoperative mortality in cardiac surgery patients | | | |
| --- | --- | --- | --- |
| Group | HR | 95% CI | *p*-value |
| Non | 1 | - | - |
| Discontinued 1 day preoperatively | 1.016 | 0.844–1.223 | 0.865 |
| Discontinued on day of surgery | 0.666 | 0.507–0.875 | 0.004 |
| Continued through surgery | 0.770 | 0.592–1.002 | 0.051 |
| ACEIs, Angiotensin-converting enzyme inhibitors; CI, confidence interval; HR, hazard ratio. | | | |
