# Supplementary material for: The impact of dose and discontinuation timing of preoperative ACE inhibitors on survival outcomes in cardiac surgery: A MIMIC-IV database analysis
Source: PLoS One. 2025 Nov 10;20(11):e0334889. doi: 10.1371/journal.pone.0334889 (PMC12599911; doi:10.1371/journal.pone.0334889)
Supplement: S6 Table — (DOCX) [file pone.0334889.s006.docx]

| **Table S6** Dose-response relationship between preoperative lisinopril use and hospital mortality in cardiac surgery patients | | | |
| --- | --- | --- | --- |
| Dose | HR | 95% CI | *p*-value |
| Non | 1 | - | - |
| ＜10 | 0.729 | 0.534–0.995 | 0.047 |
| 10-20 | 0.480 | 0.301–0.765 | 0.002 |
| ≥20 | 0.554 | 0.388–0.791 | 0.001 |
| CI, confidence interval; HR, hazard ratio. | | | |
