# Supplementary material for: The impact of dose and discontinuation timing of preoperative ACE inhibitors on survival outcomes in cardiac surgery: A MIMIC-IV database analysis
Source: PLoS One. 2025 Nov 10;20(11):e0334889. doi: 10.1371/journal.pone.0334889 (PMC12599911; doi:10.1371/journal.pone.0334889)
Supplement: S8 Table — (DOCX) [file pone.0334889.s008.docx]

| **Table S8** Dose-response relationship between preoperative lisinopril use and 90-day mortality in Cardiac Surgery Patients | | | |
| --- | --- | --- | --- |
| Dose | HR | 95% CI | *p*-value |
| Non | 1 | - | - |
| ＜10 | 0.926 | 0.739–1.160 | 0.503 |
| 10-20 | 0.660 | 0.473–0.922 | 0.015 |
| ≥20 | 0.737 | 0.576–0.944 | 0.016 |
| CI, confidence interval; HR, hazard ratio. | | | |
