# Supplementary material for: The impact of dose and discontinuation timing of preoperative ACE inhibitors on survival outcomes in cardiac surgery: A MIMIC-IV database analysis
Source: PLoS One. 2025 Nov 10;20(11):e0334889. doi: 10.1371/journal.pone.0334889 (PMC12599911; doi:10.1371/journal.pone.0334889)
Supplement: S9 Table — (DOCX) [file pone.0334889.s009.docx]

| **Table S9** Dose-response relationship between preoperative lisinopril use and 360-day mortality in Cardiac Surgery Patients | | | |
| --- | --- | --- | --- |
| Dose | HR | 95% CI | *p*-value |
| Non | 1 | - | - |
| ＜10 | 0.997 | 0.830–1.197 | 0.972 |
| 10-20 | 0.740 | 0.568–0.964 | 0.026 |
| ≥20 | 0.851 | 0.702–1.031 | 0.100 |
| CI, confidence interval; HR, hazard ratio. | | | |
