# Supplementary material for: The impact of dose and discontinuation timing of preoperative ACE inhibitors on survival outcomes in cardiac surgery: A MIMIC-IV database analysis
Source: PLoS One. 2025 Nov 10;20(11):e0334889. doi: 10.1371/journal.pone.0334889 (PMC12599911; doi:10.1371/journal.pone.0334889)
Supplement: S10 Table — (DOCX) [file pone.0334889.s010.docx]

| **Table S10** Association between preoperative use of ACEIs and all-cause mortality after propensity score matching | | | | | | |
| --- | --- | --- | --- | --- | --- | --- |
| Variables | Model 1 | | Model 2 | | Model 3 | |
|  | HR (95% CI) | *p*-value | HR (95% CI) | *p*-value | HR (95% CI) | *p*-value |
| In-hospital mortality |  | | | | | |
| ACEIs vs. Non-ACEIs | 0.677 (0.558, 0.821) | <0.001 | 0.646 (0.532, 0.784) | <0.001 | 0.635 (0.522, 0.772) | <0.001 |
| 30-day mortality |  | | | | | |
| ACEIs vs. Non-ACEIs | 0.675 (0.554, 0.822) | <0.001 | 0.658 (0.540, 0.802) | <0.001 | 0.675 (0.553, 0.825) | <0.001 |
| 90-day mortality |  | | | | | |
| ACEIs vs. Non-ACEIs | 0.706 (0.590, 0.843) | <0.001 | 0.691 (0.578, 0.826) | <0.001 | 0.717 (0.598, 0.858) | <0.001 |
| 360-day mortality |  | | | | | |
| ACEIs vs. Non-ACEIs | 0.736 (0.624, 0.869) | <0.001 | 0.715 (0.606, 0.844) | <0.001 | 0.750 (0.634, 0.887) | <0.001 |
| Model 1: Unadjusted.  Model 2: Adjusted for gender, age, race.  Model 3: Adjusted for gender, admission age, race, anion gap, bun, glucose, scr, cancer, rr, chronic kidney disease, diabetes mellitus, temperature, myocardial infarction, hr, heart failure, essential hypertension, hyperlipidemia.  ACEIs, Angiotensin-converting enzyme inhibitors; CI, confidence interval; HR, hazard ratio. | | | | | | |
